# Case report: Harel–Yoon syndrome (*ATAD3A* p.Arg528Trp) with relative cognitive preservation despite severe motor impairment

**DOI:** 10.3389/fgene.2026.1881863

**Published:** 2026-10-19

**Authors:** Paula Nathalia Zamudio Soledad, Juan Sebastián Montoya, David Santiago Poveda-Rodriguez, Luz Mey King Chio Cho, Marco Aurelio Reyes, Oscar Bernal Pacheco

**Affiliations:** Department of Neurology, Hospital Militar Central, Universidad Militar Nueva Granada, Bogotá, Colombia

**Keywords:** ATAD3A, case report, genotype–phenotype correlation, Harel–Yoon syndrome, mitochondrial disease

## Abstract

**Background:**

Harel–Yoon syndrome (HAYOS) is a rare neurometabolic disorder caused by pathogenic variants in the *ATAD3A* gene, a key regulator of mitochondrial dynamics and cellular homeostasis. Although a broad phenotypic spectrum has been described, the relationship between genotype and long-term functional outcomes remains incompletely understood.

**Case Presentation:**

We report two adult sisters with genetically confirmed HAYOS who exhibited early-onset hypotonia progressing to spasticity, craniofacial dysmorphism, optic atrophy, and sensorimotor polyneuropathy. Neuroimaging revealed mild cerebellar atrophy, and electrophysiological studies showed a chronic sensorimotor polyneuropathy with axonal loss and conduction slowing. Initial exome sequencing revealed a variant of uncertain significance in *COL5A1*, whereas subsequent targeted familial Sanger sequencing confirmed the heterozygous *ATAD3A* NM_001170535.3:c.1582C>T (p.Arg528Trp) variant in both sisters. Parental segregation could not be assessed because their father died before genetic evaluation and their mother declined further clinical evaluation and targeted segregation testing for the familial *ATAD3A* variant. Despite severe progressive motor impairment leading to wheelchair dependence, both patients completed formal education and had low-average IQ scores (88 and 82) at single adult assessments.

**Conclusion:**

These cases expand the phenotypic spectrum of HAYOS by demonstrating that severe progressive motor disability may coexist with relative cognitive preservation at adult assessment, limited cardiac or metabolic involvement, and long-term survival. Our findings highlight the diagnostic challenges posed by the ATAD3 locus and underscore the importance of comprehensive genetic evaluation in suspected mitochondrial disorders. Rather than representing a globally mild phenotype, these patients show a motor-severe but cognitively and systemically attenuated presentation, with implications for prognosis, genetic counseling, and clinical management.

## 1 Introduction

Harel–Yoon syndrome (HAYOS) is a rare neurometabolic disorder caused by pathogenic *ATAD3A* variants and marked by substantial phenotypic heterogeneity. Presentations range from lethal neonatal encephalopathy and cardiomyopathy to longer-surviving neurological phenotypes. Hypotonia, progressive spasticity, optic atrophy, and peripheral neuropathy are recurrent features, but neurological and systemic involvement varies (AlAyed et al., 2020; Harel et al., 2016). Disease severity reflects inheritance, allelic effect, and the structural context of the complex ATAD3 locus.

Adults with severe motor impairment and relative cognitive preservation are rarely described. We report two adult sisters with HAYOS carrying *ATAD3A* p.Arg528Trp who show profound motor disability but relative cognitive preservation, extending current genotype–phenotype and long-term outcome data.

## 2 Case description

Two sisters, aged 37 and 26 years, born to first-cousin parents after uneventful pregnancies and normal perinatal periods, were evaluated for progressive neurological impairment (Figure 1). Family history did not identify other relatives with known neurodevelopmental or neurological disorders. Their father died before genetic evaluation, and their mother declined further clinical evaluation and targeted segregation testing for the familial *ATAD3A* variant; therefore, parental phenotyping and segregation of *ATAD3A* could not be formally assessed. Available developmental and educational milestones are summarized in Figure 2. Exact ages at loss of independent ambulation, onset of optic atrophy, electrophysiological diagnosis of neuropathy, genetic diagnosis, and cognitive testing could not be established from the retrospective records.

**FIGURE 1:**
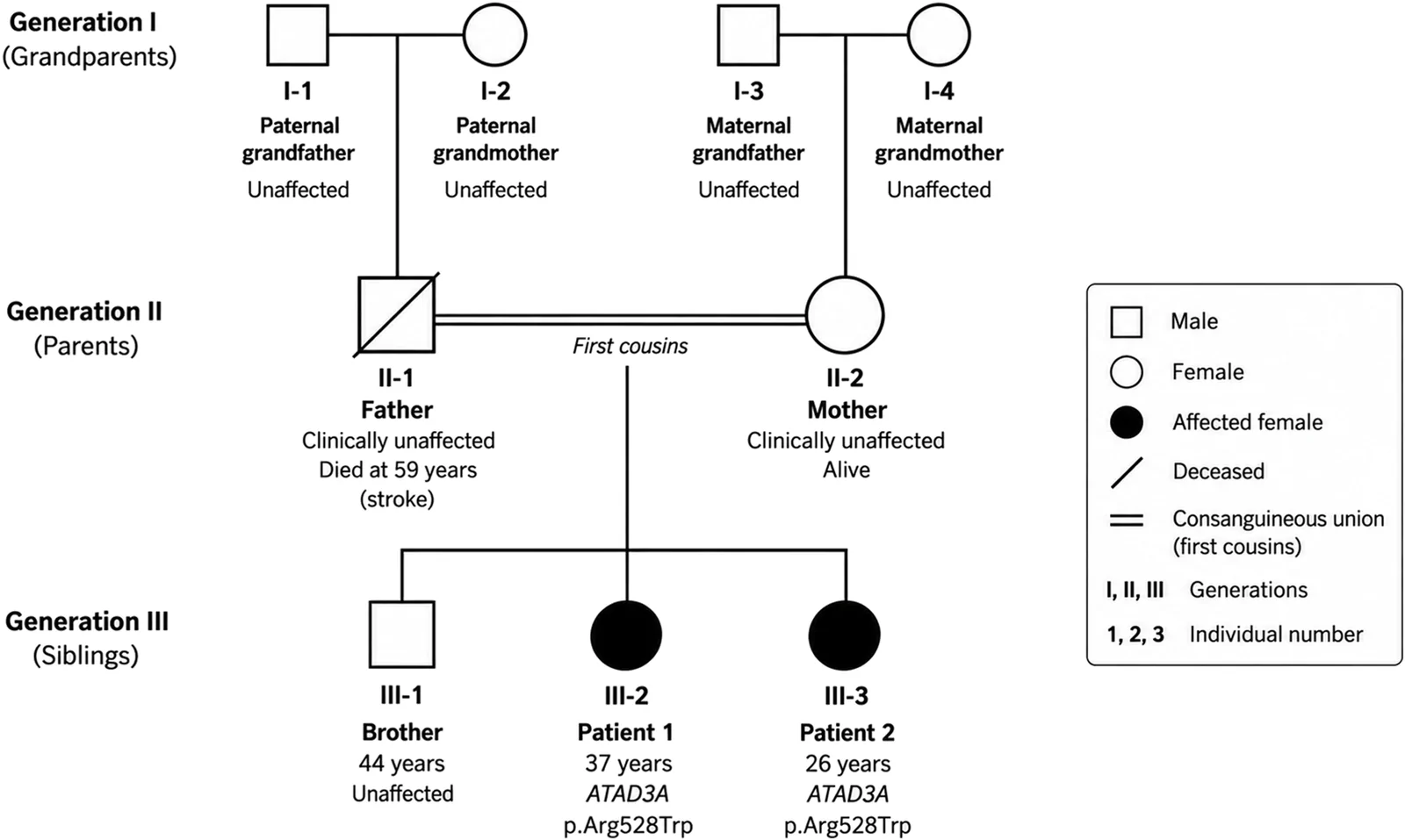
Family pedigree of two sisters with *ATAD3A*-related Harel–Yoon syndrome. Three-generation pedigree showing two affected sisters harboring the heterozygous *ATAD3A* NM_001170535.3:c.1582C>T (p.Arg528Trp) variant. The parents were reported to be first cousins; however, the specific familial relationship between individuals in generation I could not be established from the available family history. Both parents were reportedly neurologically unaffected based on family history; neither underwent targeted segregation testing for the familial *ATAD3A* variant. The father died at 59 years of age from a stroke before genetic evaluation, and the mother declined further clinical evaluation and targeted *ATAD3A* segregation testing. One older brother (44 years old) is reportedly neurologically unaffected. Labels of unaffected status in the pedigree reflect family report rather than formal evaluation. No additional family members with neurodevelopmental delay, progressive motor impairment, optic atrophy, neuropathy, epilepsy, cardiomyopathy, early unexplained deaths, miscarriages, or stillbirths were reported. Segregation and the mode of inheritance in this family therefore remain unresolved. All four grandparents were reportedly unaffected.

**FIGURE 2:**
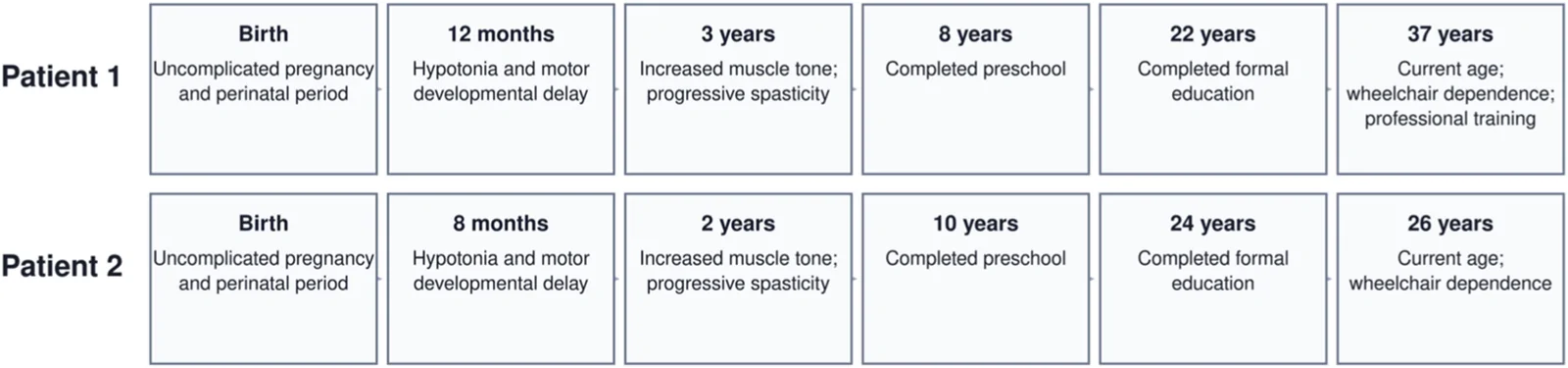
Clinical timeline of documented developmental, motor, educational, and vocational milestones in both sisters.

### 2.1 Patient 1

A 37-year-old woman presented with a history of early motor developmental delay, characterized by hypotonia beginning at 12 months of age; increased muscle tone and progressive spasticity were evident by 3 years. No clear evidence of global cognitive delay was documented. She completed preschool at 8 years, formal education at 22 years, and professional training at 37 years. Her delayed educational trajectory was likely influenced substantially by severe motor limitations, although the relative contributions of motor, cognitive, and educational factors cannot be determined retrospectively. A single cognitive assessment in adulthood yielded a reported intelligence quotient (IQ) of 88, within the low-average range.

On neurological examination, she exhibited marked lower-limb hypotrophy, generalized spasticity, bradykinesia, and scanning dysarthria (Figures 3D,E). Craniofacial features included blepharoptosis at rest (Figure 3A), gingival hypertrophy (Figure 3B), a high forehead (Figure 3C), mandibular protrusion, and low-set ears. Ophthalmological evaluation revealed optic atrophy and early cataracts. A nasal voice was also noted.

**FIGURE 3:**
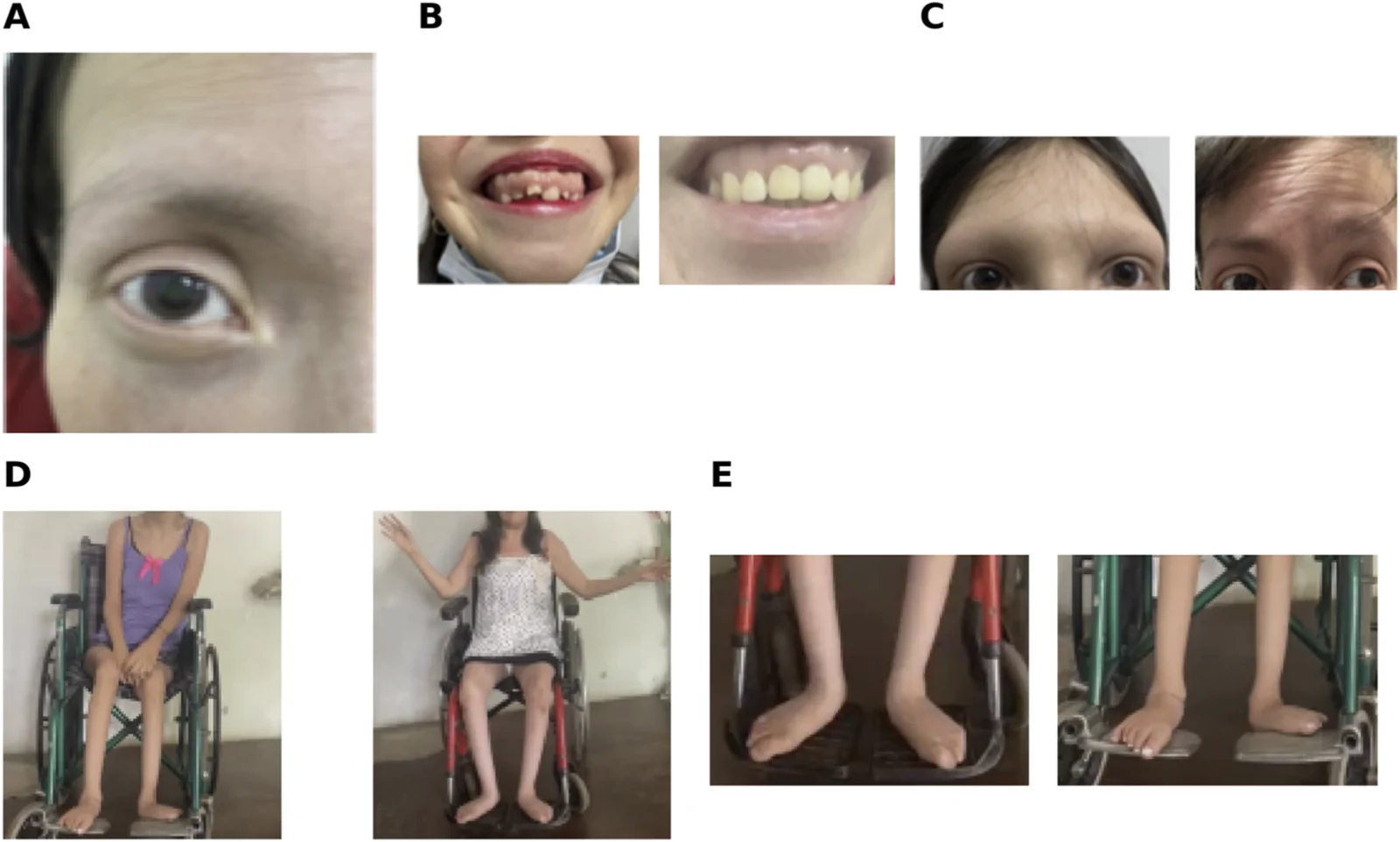
Clinical features of the two sisters. **(A)** Resting blepharoptosis in Patient 1. **(B)** Gingival hypertrophy in both patients. **(C)** Prominent forehead in both patients. **(D)** Seated clinical photographs showing wheelchair dependence and marked lower-limb hypotrophy. **(E)** Distal lower-limb photographs showing bilateral foot deformities.

Spasticity, initially graded as 4 on the Modified Ashworth Scale, improved to 2–3 following treatment with botulinum toxin injections. This was associated with improved ease of passive limb mobilization and facilitation of basic caregiving activities, including hygiene and positioning.

Functional assessment using the Barthel Index showed a score of 45/100, consistent with moderate-to-severe dependency in activities of daily living.

### 2.2 Patient 2

A 26-year-old woman presented with hypotonia beginning at 8 months of age, followed by progressive spasticity from 2 years of age. Motor impairment worsened over time and ultimately resulted in loss of independent ambulation and wheelchair dependence. Despite this progression, she completed preschool at 10 years and formal education at 24 years. Her delayed educational trajectory was likely influenced by severe motor disability and reduced mobility; however, the relative contributions of motor, cognitive, and educational factors cannot be determined retrospectively. A single cognitive assessment in adulthood yielded a reported intelligence quotient (IQ) of 82, within the low-average range.

For both sisters, the retrospective records did not specify whether education was mainstream or special, whether individualized educational support was provided, or their current literacy and communication skills. Beyond the professional training documented for Patient 1, occupational and vocational activities were not systematically recorded. The cognitive records did not identify the test or version, precise age at administration, examiner’s professional qualification, verbal/performance or nonverbal indices, confidence intervals, or adaptations for motor impairment, dysarthria, optic atrophy, or slowed response speed. Serial neuropsychological data were unavailable; therefore, relative cognitive preservation refers to cross-sectional adult performance compared with previously reported p.Arg528Trp cases, not to demonstrated longitudinal stability.

Neurological examination showed generalized spasticity, lower-limb hypotrophy, bradykinesia, and ataxic dysarthria (Figures 3D,E). Craniofacial features were consistent with those observed in her sister, including gingival hypertrophy (Figure 3B), a high forehead (Figure 3C), mandibular protrusion, and low-set ears. Optic atrophy was present, without evidence of cataracts.

Spasticity was severe on the Modified Ashworth Scale and showed a clinically meaningful reduction following targeted therapy. She is undergoing periodic botulinum toxin injections in combination with ongoing physical and multidisciplinary rehabilitation, which has facilitated passive limb mobilization and improved the ease of caregiving activities, including hygiene and positioning, despite persistent motor impairment.

Functional evaluation using the Barthel Index showed a score of 30/100, indicating severe dependency in activities of daily living.

### 2.3 Ancillary studies

Brain magnetic resonance imaging (MRI) performed in both patients demonstrated mild diffuse cerebellar atrophy, without evidence of supratentorial cortical atrophy, white matter abnormalities, basal ganglia involvement, or structural brain malformations. Electrophysiological studies were clinically interpreted as showing a chronic sensorimotor polyneuropathy with axonal loss and conduction slowing affecting all four limbs. Individual nerve-conduction values were unavailable in the source records, precluding retrospective assessment against formal demyelination criteria; some slowing may therefore have been secondary to axonal loss.

Electroencephalography showed no epileptiform activity. Cardiac evaluation, including echocardiography, was unremarkable, and metabolic screening was within normal limits.

### 2.4 Genetic testing

Clinical duo whole-exome sequencing (WES) was performed in 2023 using genomic DNA obtained from peripheral blood. Next-generation sequencing was performed on a NextSeq platform, targeting approximately 19,000 genes, and bioinformatic interpretation was conducted using QIAGEN Digital Insights. The laboratory reported that all disclosed variants had a read depth greater than 20×. Synonymous variants, variants with a population allele frequency greater than 1%, and variants classified as benign or likely benign were excluded from reporting.

The WES report identified the heterozygous *COL5A1* NM_000093.5:c.4252C>T (p.Pro1418Ser) variant, classified by the laboratory as a variant of uncertain significance, and the heterozygous *EPM2A* NM_005670.4:c.721C>T (p.Arg241*) variant, classified as pathogenic in association with an autosomal recessive disorder. Both variants were described in the laboratory report as maternally inherited. Because only one *EPM2A* allele was affected, this finding represented carrier status and did not explain the neurological phenotype. The variants disclosed in the available WES report are summarized in Supplementary Table S1.

Neither affected sister showed clinical findings suggestive of a *COL5A1*-related disorder, including skin hyperextensibility, abnormal scarring, easy bruising, recurrent joint dislocations, or generalized joint hypermobility. Furthermore, the neurological phenotype, characterized by progressive spasticity, optic atrophy, and sensorimotor neuropathy, was not consistent with *COL5A1*-associated disease. Therefore, the *COL5A1* variant was considered unlikely to explain the neurological presentation and was interpreted as an incidental finding. Neither the *COL5A1* nor the heterozygous *EPM2A* variant has an established protective or modifier role in *ATAD3A*-related disease.

The clinical indication documented in the WES report stated that the heterozygous *ATAD3A* NM_001170535.3:c.1582C>T (p.Arg528Trp) variant had previously been identified in an affected sister. However, this variant was not included among the findings reported by WES. Because the original sequencing files and locus-specific coverage data were unavailable for retrospective review, the reason why the variant was not reported could not be determined. Accordingly, it is described as “not reported by the initial WES” rather than “not detected.”

Given the clinical suspicion of a mitochondrial disorder, the known familial variant, and the closely concordant phenotype of both sisters, targeted familial testing was subsequently performed. This was not a multigene panel but single-site Sanger sequencing specifically directed at *ATAD3A* NM_001170535.3:c.1582C>T (p.Arg528Trp). Genomic DNA was extracted from peripheral blood using the High Pure PCR Template Preparation Kit (Roche). Sanger sequencing was performed using the BigDye Direct Cycle Sequencing Kit on a SeqStudio Genetic Analyzer, and sequence data were analyzed using Variant Reporter software (Thermo Fisher Scientific). The variant was confirmed in heterozygosity in both affected sisters, supporting the diagnosis of an *ATAD3A*-related Harel–Yoon syndrome phenotype.

Variant pathogenicity was reassessed using primary evidence within the ACMG/AMP framework (Richards et al., 2015). The *ATAD3A* p.Arg528Trp substitution has been reported as *de novo* in five unrelated affected individuals, and experimental studies demonstrated abnormal mitochondrial morphology, reduced mitochondrial content, and increased mitophagy in a *Drosophila* model, together with increased mitophagy in patient fibroblasts (Harel et al., 2016). ClinVar lists the variant as pathogenic/likely pathogenic, with two submissions and no conflicts (RCV000488909.5) (National Center for Biotechnology Information, 2026). Applying PS2 (recurrent *de novo* occurrence), PS3 (supportive functional evidence), PM2 (absence or extreme rarity in population databases), and PP3 (concordant computational evidence), we classified p.Arg528Trp as pathogenic under the ACMG/AMP framework. PM5 was not applied because the published evidence concerns the same recurrent substitution rather than a different pathogenic missense change at Arg528, and PP5 was not used as an independent evidence criterion.

## 3 Discussion

We report two adult sisters harboring the heterozygous *ATAD3A* c.1582C>T (p.Arg528Trp) variant who developed a severe, progressive, motor-predominant neurological disorder with comparatively mild cognitive involvement at adult assessment. Both patients had infantile hypotonia followed by progressive spasticity, lower-limb hypotrophy, dysarthria, loss of independent ambulation, optic atrophy, mixed sensorimotor polyneuropathy, and mild cerebellar atrophy. Despite Barthel Index scores of 45/100 and 30/100, they survived into their fourth and third decades, completed formal education, and obtained IQ scores of 88 and 82. Their phenotype is therefore not globally mild: motor disability is profound, whereas cognitive involvement, systemic disease, and effect on survival are comparatively attenuated.

The patient-level literature for this recurrent variant is larger than the original five-case comparison. Harel et al. (2016) described five unrelated individuals with *de novo* p.Arg528Trp. Lepelley et al. (2021) later reported four carriers, but their patient 5 was explicitly identified as Harel family 3, individual II-1; therefore, that publication added three, not four, unique patients. Mei et al. (2025) searched the literature through 30 April 2024 and enumerated eight p.Arg528Trp carriers, corresponding to the five Harel patients plus the three additional Lepelley patients. The distinct AlAyed infant is absent from the supplementary patient list of that review, increasing the pre-cutoff total to at least nine (AlAyed et al., 2020). Reports published after the review cutoff added a 2-year-old girl in a Saudi neuromuscular cohort, a Chinese infant with early cardiac and metabolic decompensation, and a Chinese boy with severe developmental delay and seizures (Al-Hedaithy et al., 2025; Huang et al., 2025; Yang et al., 2025). Thus, at least 12 unique previously published individuals with p.Arg528Trp can be distinguished before the present family. Table 1 compares these patients individually and counts duplicate follow-up only once.

**TABLE 1:** Patient-level comparison of unique individuals carrying heterozygous *ATAD3A* c.1582C>T (p.Arg528Trp).

Report/case	Sex; age at last report	Inheritance	Development/cognition	Motor phenotype/peripheral nerve	Ophthalmic/brain findings	Cardiac, metabolic, and other findings	Functional status/outcome
Harel case 1 (Harel et al., 2016)	F; 9 years	*De novo*	Developmental delay; mild ID	Hypotonia, spasticity, axonal neuropathy	Optic atrophy	No HCM; metabolic data NR; ADHD	Ambulatory; alive at 9 years
Harel case 2 (Harel et al., 2016)	F; 5 years	*De novo*	Developmental delay/ID	Hypotonia, spasticity, axonal neuropathy	NR	No HCM; lactate and complex I + III abnormalities	Ambulatory; alive at 5 years
Harel case 3; Lepelley P5, duplicate follow-up (Harel et al., 2016; Lepelley et al., 2021)	M; 7 years (latest)	*De novo*	Global developmental delay/ID	Truncal hypotonia, peripheral spasticity, neuropathy	Optic nerve pallor; macular hypoplasia	HCM from 3 months; intermittent lactate elevation/MRS peak; vitiligo; GH deficiency	Alive at 7 years; detailed ambulation NR
Harel case 4 (Harel et al., 2016)	F; 5 years	*De novo*	Developmental delay/ID	Hypotonia, spasticity, axonal neuropathy	Optic atrophy	HCM; lactate, 3-MGA, and respiratory-chain abnormalities	Ambulatory; alive at 5 years
Harel case 5 (Harel et al., 2016)	M; 23 months	*De novo*	Developmental delay/ID	Hypotonia; no spasticity; borderline neuropathy	NR	No HCM; metabolic data NR; 47,XXY	Ambulation NR; alive at 23 months
Lepelley P1 (Lepelley et al., 2021)	F; 9 years	*De novo*	Profound global delay; communication limited to eyes/facial expression	Central hypotonia, rigid dystonia; no useful motor function	Visual impairment; nonspecific MRI signal changes	Refractory seizures; elevated ISG; cardiac/metabolic data NR	No useful motor function; alive at 9 years
Lepelley P3 (Lepelley et al., 2021)	F; 4 years	*De novo*	Severe motor delay; impaired cognition; good social contact	Severe flexion contractures; peripheral nerve findings NR	NR	Systemic sclerosis; feeding difficulty/gastrostomy; normal fibroblast respiratory-chain assay; elevated ISG	Alive at 4 years
Lepelley P4 (Lepelley et al., 2021)	M; 2 years	*De novo*	Severe developmental delay	Central hypotonia, peripheral dystonia, neuropathy	Cranial CT: no calcification; other findings NR	HCM; complex I and V deficiency; cerebral MRS lactate peak; elevated ISG/IFN-alpha	Alive at 2 years
AlAyed et al. (AlAyed et al., 2020)	F; 20 months	*De novo*	Severe global delay; no meaningful milestones or speech	Axial hypotonia, appendicular hypertonia; normal NCS	Optic/visual impairment; mild cerebral atrophy; delayed myelination; severe SNHL	Normal echo; lactate 4 mmol/L; 3-MGA; failure to thrive	Never rolled or sat; alive at 20 months
Al-Hedaithy case 46 (Al-Hedaithy et al., 2025)	F; 2 years	NR	Motor delay; cognition NR	Motor delay; peripheral nerve findings NR	NR	NR	Outcome and detailed function NR
Huang et al. (Huang et al., 2025)	F; ∼2 months	*De novo*	Poor mental status; development not assessable in early infancy	Slight hypotonia; peripheral nerve findings NR	Cranial ultrasound normal	Cardiac insufficiency/noncompaction-hypertrophic phenotype; lactate 19.8 mmol/L; hyperammonemia; feeding difficulty, cyanosis, stridor	Discharged after recurrent admissions; later outcome NR
Yang et al. (Yang et al., 2025)	M; 3 years 10 months	*De novo*	Severe global developmental delay	Axial hypotonia; motor delay	Facial dysmorphism; ophthalmic/brain findings NR	Epileptic spasms and myoclonic seizures; cardiac/metabolic data NR	Seizures controlled and motor function improved with treatment
Present case 1	F; 37 years	Heterozygous; inheritance unresolved; parents not formally evaluated or tested	Single adult IQ 88, low-average; test details NR; completed formal education and professional training	Infantile hypotonia; severe progressive spastic paraparesis; mixed sensorimotor polyneuropathy; wheelchair	Optic atrophy; mild cerebellar atrophy; early cataracts	No seizures or HCM; normal metabolic evaluation	Alive at 37 years; Barthel Index 45/100
Present case 2	F; 26 years	Heterozygous; inheritance unresolved; parents not formally evaluated or tested	Single adult IQ 82, low-average; test details NR; completed formal education	Infantile hypotonia; severe progressive spastic paraparesis; mixed sensorimotor polyneuropathy; wheelchair	Optic atrophy; mild cerebellar atrophy; no cataracts	No seizures or HCM; normal metabolic evaluation	Alive at 26 years; Barthel Index 30/100

Unique individuals only; Lepelley patient 5 is Harel case 3. Mei et al. (2025) counted eight p.Arg528Trp carriers through 30 April 2024 (five Harel plus three additional Lepelley cases). NM_018188.5:c.1726C>T (p.Arg576Trp) and NM_001170535.3:c.1582C>T (p.Arg528Trp) are transcript-equivalent descriptions of the same variant.

ADHD, attention-deficit/hyperactivity disorder; GH, growth hormone; HCM, hypertrophic cardiomyopathy; ID, intellectual disability; IFN, interferon; ISG, interferon-stimulated gene; 3-MGA, 3-methylglutaconic aciduria; MRS, magnetic resonance spectroscopy; NCS, nerve-conduction studies; NR, not reported; SNHL, sensorineural hearing loss.

In the original Harel cohort, the five patients were evaluated between 23 months and 9 years of age (Harel et al., 2016). All had early developmental delay and hypotonia; four developed spasticity, four had definite peripheral neuropathy and the fifth had borderline electrophysiological findings, three had optic atrophy, and two had hypertrophic cardiomyopathy. Lactate elevation and respiratory-chain abnormalities were variable. Four retained independent ambulation at their last pediatric assessment, whereas this information was unavailable for the youngest patient. Follow-up of Harel case 3 as Lepelley patient 5 documented persistent global developmental delay, truncal hypotonia, peripheral spasticity and neuropathy, optic nerve pallor, and hypertrophic cardiomyopathy at 7 years (Lepelley et al., 2021). However, the exclusively pediatric follow-up precluded evaluation of motor progression over subsequent decades.

The three additional unique patients reported by Lepelley et al. (2021) were also severely affected children but illustrated substantial phenotypic variability. Patient 1 had profound developmental impairment, refractory seizures, visual dysfunction, and no useful motor function; patient 3 had impaired cognition, gastrostomy-dependent feeding, contractures, and systemic sclerosis; and patient 4 had severe developmental delay, neuropathy, hypertrophic cardiomyopathy, respiratory-chain deficiency, and cerebral lactate elevation. Other reports further expanded this spectrum. AlAyed et al. (2020) described severe developmental delay, optic atrophy, hearing loss, and metabolic abnormalities but normal nerve-conduction and cardiac studies. Only limited phenotypic information was available for the 2-year-old patient described by Al-Hedaithy et al. (2025). Huang et al. (2025) described neonatal cardiac and metabolic decompensation, whereas Yang et al. (2025) reported severe developmental delay and early-onset seizures. Collectively, previously reported p.Arg528Trp patients were children with predominantly severe neurodevelopmental disease and variable cardiac, metabolic, epileptic, sensory, or inflammatory involvement.

Our sisters share the core p.Arg528Trp manifestations of infantile hypotonia, pyramidal involvement, optic atrophy, and peripheral neuropathy, but their clinical trajectory differs in several directly observed respects. At ages 26 and 37 years, both had severe progressive spastic paraparesis, wheelchair dependence, and marked functional dependency, yet their single adult IQ scores were in the low-average range and both completed formal education. Neither had seizures, cardiomyopathy, lactate elevation, or other detected metabolic abnormalities, and MRI showed only mild cerebellar atrophy without major supratentorial structural disease. No previously published p.Arg528Trp patient in Table 1 was followed into adulthood; consequently, these sisters provide information about the long-term motor course that cannot be obtained from the pediatric reports.

Age is the most evidence-based explanation for the apparent difference in motor severity. The pediatric cohorts could not reveal decades of progressive spasticity, neuropathy, or eventual loss of ambulation, and the sisters' adult disability may represent age-dependent progression of the same disorder rather than a separate phenotype. Age does not, however, explain their comparatively mild cognitive involvement at adult assessment or the absence of cardiac and metabolic abnormalities. The lack of hypertrophic cardiomyopathy, metabolic decompensation, and severe feeding or respiratory disease is clinically consistent with their prolonged survival, although this case report cannot establish a causal relationship.

The IQ scores of 88 and 82 provide evidence of less cognitive involvement than in most previously reported p.Arg528Trp patients, but incomplete test documentation and severe motor, speech, and visual limitations restrict their interpretation. These single assessments do not establish entirely normal cognition or longitudinal stability. The concordant corticospinal, peripheral nerve, optic, and cerebellar involvement with limited supratentorial disease may suggest selective neural vulnerability, but this was not tested in patient-derived tissue. Likewise, the similar phenotype in both sisters raises the possibility of a shared genetic modifier, yet no modifier analysis was performed. These remain testable hypotheses rather than explanations established by the present data.

Functional evidence supports pathogenicity but does not currently explain the attenuated cognitive and systemic phenotype. Harel et al. (2016) showed that the homologous *Drosophila* variant caused abnormal mitochondrial morphology, reduced mitochondrial content, and increased mitophagy, and patient fibroblasts also showed increased mitophagy. Further work supports dominant interference within oligomeric *ATAD3A* complexes (Yap et al., 2021). Lepelley et al. (2021) additionally demonstrated mtDNA-dependent cGAS–STING activation and increased type I interferon signaling in several *ATAD3A*-mutated patients and cellular models. Neither mitochondrial functional studies nor interferon measurements were performed in our sisters, and they had no reported systemic-sclerosis phenotype; therefore, these pathways cannot be used to account for their individual clinical differences.

The recurrence of p.Arg528Trp in two sisters requires cautious genetic interpretation. Parental segregation could not be assessed because their father died before the genetic evaluation and their mother declined further clinical evaluation and targeted segregation testing for the familial *ATAD3A* variant. Parental germline or low-level somatic mosaicism remains a plausible explanation for recurrence, but inherited transmission and other familial mechanisms cannot be distinguished without parental data. Mosaicism would explain recurrence, not the attenuated cognitive or systemic phenotype. Furthermore, extensive homology among *ATAD3A*, *ATAD3B*, and *ATAD3C* limits conventional short-read detection of copy-number changes and complex rearrangements. Targeted copy-number analysis and long-read sequencing in the sisters would help exclude an additional contributor, although they would not resolve inheritance without parental samples.

Their restricted systemic involvement contrasts with the profound encephalopathy, congenital abnormalities, cardiomyopathy, respiratory failure, and early mortality associated with biallelic loss-of-function variants and large structural rearrangements of the ATAD3 locus (Harel et al., 2016). Nevertheless, severe motor disability shows that adult survival and relative cognitive preservation do not imply limited neurological severity.

No disease-modifying treatment is established, and management remains supportive. In our patients, botulinum toxin for spasticity together with rehabilitation, positioning, and multidisciplinary care improved passive mobility, comfort, hygiene, and caregiving, but did not alter disease progression.

### 3.1 Limitations

This report is limited by the study of two related individuals, the absence of patient-derived functional studies, and the lack of parental segregation. Cryptic variation within the ATAD3 region cannot be excluded, and parental mosaicism or inherited transmission remains unresolved. Comprehensive longitudinal neuropsychological data were unavailable. In addition, several published p.Arg528Trp reports provide incomplete clinical data, and differences in age and ascertainment prevent formal estimates of feature frequency or direct comparison of long-term motor outcomes.

An important limitation of this report is that the complete filtered variant list from the original WES was not available for retrospective review. The analysis was performed in 2023 at an external clinical laboratory, and only the final diagnostic report was incorporated into the clinical records available to the authors. The complete filtered dataset and the original variant call and alignment files were not transferred to the treating institution and were therefore unavailable for independent reassessment. Consequently, we could not evaluate whether additional candidate or modifier variants were present. Furthermore, locus-specific coverage and read-level data for *ATAD3A* were unavailable; therefore, the reason why the c.1582C>T (p.Arg528Trp) variant was not included among the findings reported by WES could not be established.

### 3.2 Conclusion

In conclusion, reconciliation of the literature identifies at least 12 unique previously published individuals with heterozygous *ATAD3A* p.Arg528Trp, most with severe pediatric neurodevelopmental or multisystem disease. The present sisters extend this genotype into adulthood and show that profound progressive motor disability, optic and peripheral nerve involvement, and mild cerebellar atrophy can coexist with low-average IQ scores at single adult assessments and limited cardiac or metabolic involvement. Their phenotype is best described as motor-severe but cognitively and systemically attenuated. These observations emphasize long-term follow-up, patient-level comparison without duplicate counting, comprehensive genomic evaluation, and cautious genetic counseling.

## Data Availability

The raw data supporting the conclusions of this article will be made available by the authors, without undue reservation.
