# Supplementary material for: Case report: Harel–Yoon syndrome (ATAD3A p.Arg528Trp) with relative cognitive preservation despite severe motor impairment
Source: Front Genet. 2026 Oct 19;17:1881863. doi: 10.3389/fgene.2026.1881863 (PMC13724538; doi:10.3389/fgene.2026.1881863)
Supplement: Supplementary file 1 [file Table1.docx]

Supplementary Material

# 1 Supplementary Table S1

**Supplementary Table S1.** Variants disclosed in the available clinical whole-exome sequencing report.

| **Gene** | **HGVS variant** | **Zygosity** | **Reported inheritance** | **Laboratory classification** | **Disease mechanism** | **Clinical interpretation** |
| --- | --- | --- | --- | --- | --- | --- |
| *COL5A1* | NM_000093.5:c.4252C>T (p.Pro1418Ser) | Heterozygous | Maternally inherited | Variant of uncertain significance (VUS); laboratory evidence criteria were not included in the available report | *COL5A1*-associated disorders are generally autosomal dominant | The phenotype was not consistent with a *COL5A1*-related disorder; considered incidental and not an established modifier of *ATAD3A*-related disease |
| *EPM2A* | NM_005670.4:c.721C>T (p.Arg241*) | Heterozygous | Maternally inherited | Pathogenic; laboratory evidence criteria were not included in the available report | Associated disorder is autosomal recessive | Single-allele carrier status; does not explain the neurological phenotype and is not an established modifier of *ATAD3A*-related disease |

**Note.** Only variants disclosed in the final clinical WES report are shown. The complete filtered variant list, locus-specific coverage data, and original variant call and alignment files were not available for retrospective review because the test was performed at an external clinical laboratory and only the final diagnostic report was incorporated into the clinical records available to the authors. Accordingly, this table should not be interpreted as a complete list of all variants that passed the original analytical filters.
